# Sustained effects of a multimodal campaign aiming at hand hygiene improvement on compliance and healthcare-associated infections in a large gynaecology/obstetrics tertiary-care centre in Vietnam

**DOI:** 10.1186/s13756-020-00712-x

**Published:** 2020-04-10

**Authors:** Hang Thi Phan, Walter Zingg, Hang Thi Thuy Tran, Anh Pham Phuong Dinh, Didier Pittet

**Affiliations:** 1 Infection control programme, Hung Vuong hospital, Ho Chi Minh City, Vietnam; 2 Infection control programme and WHO collaborating centre on patient safety, University of Geneva Hospitals and Faculty of Medicine, 4 Rue Gabrielle Perret-Gentil, 1205 Geneva, Switzerland

**Keywords:** Hand hygiene, Compliance, Healthcare-associated infection, Surgical site infection, Incidence, Vietnam, Lower-middle-income country, Multimodal, Behaviour change, Intervention, Alcohol-based handrub, Hand sanitizer

## Abstract

**Background:**

Hung Vuong Hospital (HVH) is a 900-bed maternity hospital in Ho-Chi-Minh-City, Vietnam. Due to low compliance, a quasi-experimental, observational study was conducted with the aim to improve hand hygiene.

**Methods:**

A multimodal promotion strategy was established in 2010 and further developed towards ongoing, repetitive and inventive campaigns including patient participation. Hand hygiene compliance was monitored by direct observation and healthcare-associated infections (HAIs) by applying standard definitions.

**Results:**

Between 2010 and 2018, a total of 43,711 hand hygiene opportunities were observed. Compliance improved from 21.5% (95%CI: 20.2–22.8%) in 2010 to 75.1% (73.9–76.2%) in 2018 (incidence rate ratio, IRR , 1.10; 95%CI, 1.10–1.11). This was achieved through increasing recourse to alcohol-based hand rubbing. A total of 554,720 women were admitted to HVH during the study period for 353,919 deliveries (198,679 vaginal; 155,240 by C-section) and 257,127 surgical procedures. The HAI-incidence decreased significantly from 1.10 episodes per 1000 patient-days in 2010 to 0.45 per 1000 patient-days in 2018 (IRR 0.85; 95%CI, 0.79–0.90). Significant improvement was observed also for surgical site infections after gynaecological surgery (IRR 0.95; 95%CI, 0.92–0.99) and endometritis after abortion (IRR 0.80; 95%CI, 0.68–0.93).

**Conclusions:**

A multimodal strategy aiming at behaviour change significantly improved and sustained hand hygiene, which contributed to the reduction of healthcare-associated infections.

## Introduction

Hand hygiene is the easiest and most effective action to prevent cross-transmission of multidrug-resistant microorganisms and healthcare-associated infections (HAIs) [1]. Multimodal promotion strategies proved to be more effective than single interventions to change healthcare workers (HCWs) behaviour [2]. Studies worldwide have shown that improvement of hand hygiene compliance contributed to HAI reduction in both acute and long-term care [3–5]. In Vietnam, similarly to landmark reports [4], research showed that the use of alcohol-based handrub (ABHR) was associated with significant decrease of surgical site infections (SSIs) in neurosurgery [6] and of HAIs in urology [7].

Enhancing hand hygiene compliance in healthcare activities improves both quality and safety of patient care. Hung Vuong Hospital (HVH) started organizing hand hygiene training in 2006. At that time, overall hand hygiene compliance averaged 32% only, and even dropped to 2 and 8% in 2008 and 2009, respectively. The aim of this prospective, quasi-experimental, observational study was to improve hand hygiene by applying an ongoing multimodal intervention strategy and to analyse its impact on HAI incidence in gynaecology and obstetrics, where the evidence-base on this subject is low.

## Methods

The report of this quasi-experimental, observational intervention study follows the “strengthening the reporting of observational studies in epidemiology” (STROBE) statement [8].

### Setting

HVH is a 900 bed-maternity referral centre in Ho Chi Minh City, Vietnam, employing around 200 physicians and 700 nurses/midwives in 16 clinical departments. On average, 40,000 new-borns were delivered each year between 2010 and 2018, 43% of them by caesarean section. This study excluded outpatient care, accident and emergency, and neonatology.

### Intervention

From 2010, the World Health Organization (WHO) multimodal hand hygiene promotion strategy with all recommended tools was implemented [9]; if not available in Vietnamese, tools were translated and adapted to the local context. In 2009, before starting the implementation of the WHO multimodal hand hygiene strategy, HVH aimed at applying a “system change” by supplying liquid soap and towels, as well as providing ABHR at the point of patient care, assuring a dispenser-to-patient ratio of 1:1 in the entire hospital. In October 2012, HVH started its own ABHR production using the WHO-ABHR formulation [10]. In 2014, video clips were produced to educate patients and relatives on the importance of hand hygiene, particularly when taking care of new-borns. Staff education started with testing hand hygiene knowledge using Vietnamese translations of the WHO questionnaires [9]. Yearly mandatory training courses were organized for new healthcare professionals. Since 2014, regular training workshops were organized in delivery wards and surgery departments. They included six activities: 1) a 10-min video outlining the reasons for hand hygiene; 2) focus group discussions about the reasons for hand hygiene; 3) a role-playing game where participants could visualise adequate hand hygiene technique with ultra-violet light; 4) focus group discussions to determine the 5 moments of hand hygiene; 5) practice of the correct hand hygiene technique following the WHO six steps; and 6) a lecture on the efficacy of alcohol-based hand-rub compared to water and soap. Between 2012 and 2013, 530 nurses and midwives were trained in 14 training sessions on aseptic technique for the insertion of peripheral venous catheters and urinary catheters. WHO posters about the role of the “5 Moments for hand hygiene” [11–13] in the prevention of catheter-associated bloodstream infection [14] and catheter-associated urinary tract infection [14] were translated into Vietnamese, and displayed in the wards.

To facilitate implementation, the promotion activities used awareness raising, evidence-based recall, continuous exposure to reminders, and actions to reinforce the institutional safety culture for hand hygiene: evidence that hand hygiene reduced childbed fever in the historical case-story of Ignaz Semmelweis; measuring bacterial hand contamination (pre/post bacterial sampling on HCWs’ hands) during patient care and after hand hygiene; translated and adapted WHO slides and other materials to support evidence-based hand hygiene guidelines and staff behavioural change; locally developed posters used as workplace reminders, as proposed by WHO [14], displayed in all departments; and internal benchmarking of hand hygiene compliance between wards.

The infection prevention and control (IPC) department at HVH participated actively to the WHO 5th May “*SAVE LIVES: Clean Your Hands”* campaign every year since 2010 [12]. Each year, different activities were offered to staff on this day, including a festival with hand hygiene competitions, staff knowledge (about hand hygiene), and serious games and poster design contests. Modest prizes were awarded to the winning HCWs, wards, or departments. Hand hygiene “how-to-handrub”-related dance performances were organized on several occasions.

The hospital director, all members of the board of directors, all heads of the departments and chiefs of nursing demonstrated their commitment to hand hygiene by signing pledges on posters, which had been designed by each department. The signed posters were transformed to screensavers, and then displayed on all computers at HVH.

### Interventions other than hand hygiene

A promotion strategy aiming at the prevention of catheter-associated urinary tract infection (CAUTI) was conducted in 2012 and 2013. Apart from hand hygiene, the strategy also included education and training on catheterisation, the distribution of sterile catheter insertion packs, and reminders at the work place. From September 2016, the WHO guidelines on SSI prevention were introduced and staff was trained on correct surgical hand preparation. Regular audits with feedback were performed on surgical hand preparation, skin disinfection, and crowding in the operating room. In addition, lectures on standard precaution measures, occupational exposure risks and waste disposal were organised once a year for all staff.

#### Audit and feedback

##### Hand hygiene monitoring

Monitoring and performance feedback of hand hygiene compliance was conducted as recommended by WHO [13] through systematic direct observation sessions [11, 15]. Auditors, six IPC professionals (3 nurses, 3 public health bachelors), were trained on the WHO material, completed the WHO online-training course, and received certification on hand hygiene monitoring by Hand Hygiene Australia [16]. Hand hygiene audits started at least 1 month before the 5th of May hand hygiene campaigns and lasted 6 months thereafter. Nurses/midwives, physicians, nursing assistants, and students with patient contact were observed. Departments and the schedules of auditing were assigned randomly to the auditors. No more than 3 nurses were allowed to be observed simultaneously, and sessions did not exceed 30 min. The results of the audits were shared with the HCWs directly after the sessions before leaving the wards. Non-compliance was reported to the supervisors. Summary results of hand hygiene compliance were reported to the hospital management and directors of the departments at regular board meetings. The department with the highest hand hygiene compliance was awarded.

##### Surveillance of healthcare-associated infections

Hospital-wide HAI surveillance started in June 2010, and was performed prospectively and without interruption over the following years. All admitted patients were eligible for surveillance, starting at day of admission until day of discharge. Surveillance was carried out by trained IPC nurses, using the definitions issued by the US Centers for Disease Prevention and Control (CDC) [17]. Charts from patients receiving antibiotics for more than 1 day were screened for HAI. Data collection was performed electronically. All HAIs were discussed with obstetricians, gynaecologists, and infectious diseases specialists. All confirmed cases were kept in an electronic file for analysis. A monthly report on HAIs as well as the results of a competency assessment of aseptic techniques was sent to the head physicians and chief of nursing of each clinical department. Perioperative antibiotic prophylaxis for SSI prevention had been introduced in 2008. The aim was to prescribe a single antimicrobial dose within 30 min before surgery. Compliance with applying one dose versus two or more doses was observed using electronic data. Compliance increased from 85% in 2008 to 92% in 2018.

##### Statistical analysis

Three primary outcomes were defined as: 1) hand hygiene compliance, obtained by direct observation and reported as the proportion of performed hand hygiene actions as per hand hygiene opportunities; 2) SSIs, reported as the proportion of infections as per surgical intervention; and 3) HAI incidence-density. Patients were followed up from admission to discharge. Trends of hand hygiene compliance (by calendar year), HAI incidence density (by calendar year), and SSI incidence proportions (by calendar year) were studied across time using Poisson regression analysis and reported as incidence rate ratios (IRR) with 95% confidence intervals (95%CI). All statistical analyses were performed using Stata, version 13.0 (StataCorp).

## Results

Between 2010 and 2018, a total of 43,711 hand hygiene opportunities were observed in 3354 sessions, with a median of 12 (interquartile range, IQR, 7–18) hand hygiene opportunities per session. Median time per session was 15 (IQR, 10–23) minutes; the total observation time was 950 h and 37 min. The 43,711 hand hygiene opportunities covered 53,421 hand hygiene indications: 9054 (20.7%) before touching a patient; 16,792 (38.4%) before clean/aseptic procedure; 15,525 (35.5%) after body fluid exposure risk; 9282 (21.2%) after touching a patient; and 2768 (6.3%) after touching patient surroundings.

**Fig. 1:**
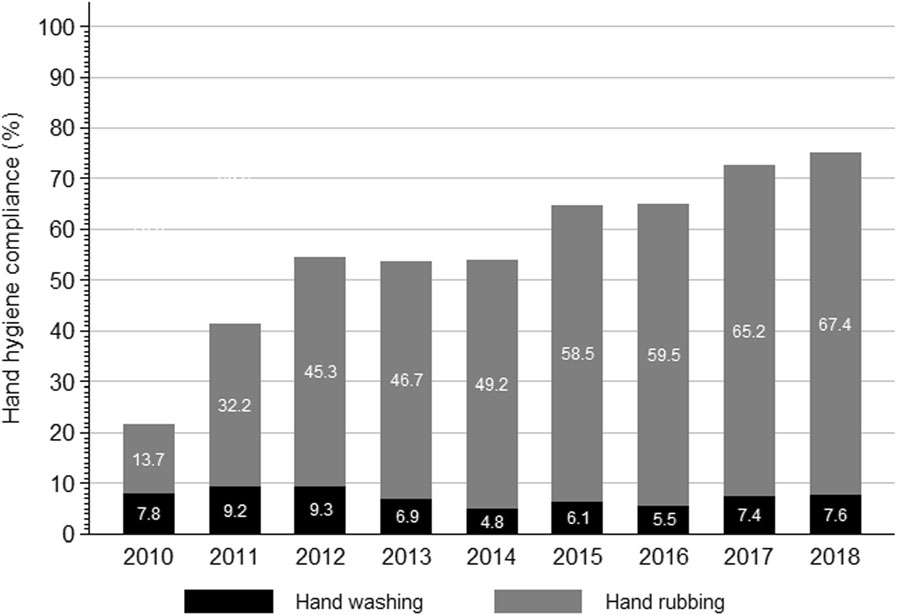
Hand hygiene compliance stratified by hand washing and hand rubbing – Hung Vuong Hospital, Ho Chi Minh City, Vietnam; 2010–2018

**Fig. 2:**
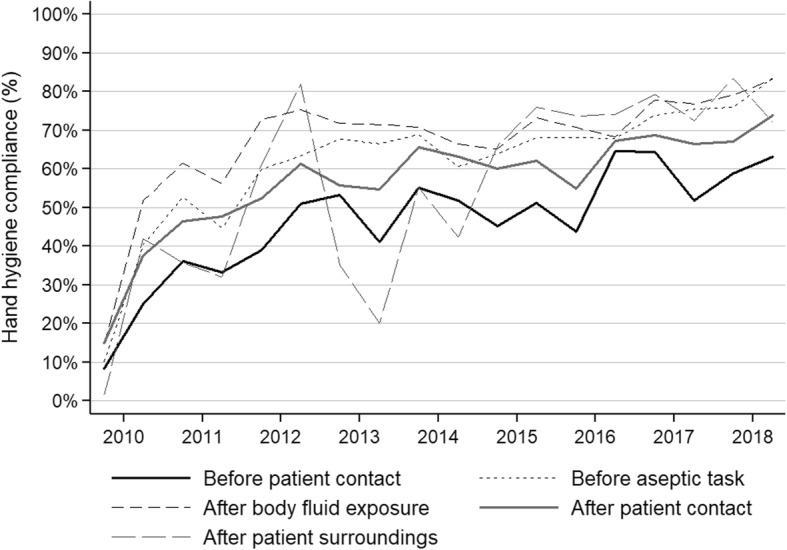
Trends of hand hygiene compliance by semester and hand hygiene indications – Hung Vuong Hospital, Ho Chi Minh City, Vietnam; 2010–2018

**Fig. 3:**
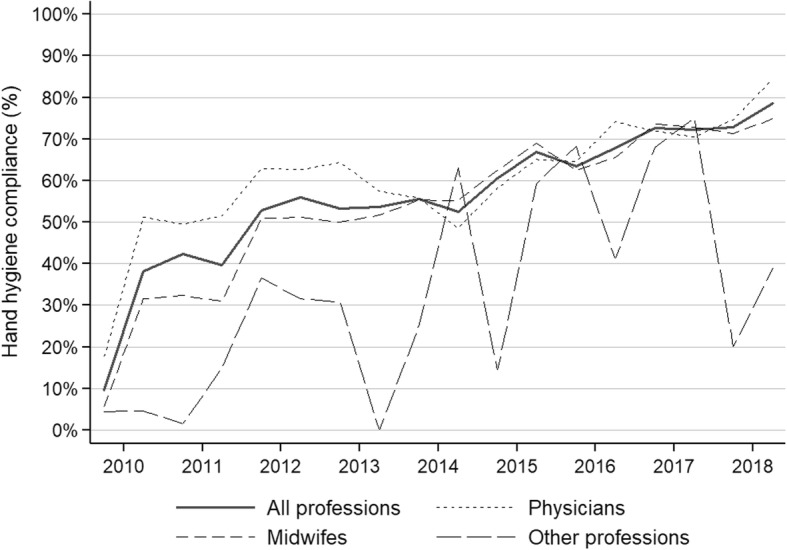
Trends of hand hygiene compliance by semester and professions – Hung Vuong Hospital, Ho Chi Minh City, Vietnam; 2010–2018

Hand hygiene compliance (either hand washing or hand rubbing) improved from 21.5% (95%CI, 20.2–22.8%) in 2010 to 75.1% (95%CI, 73.9–76.2%) in 2018 (Table 1). The trend towards improvement was significant (IRR 1.10; 95%CI , 1.10–1.11). Hand hygiene improvement was achieved through a marked increase of hand rubbing, while hand washing remained stable (Fig. 1). Compliance was highest for the indication “after body fluid exposure risk” (69.1%; 95%CI, 68.4–69.8%), followed by “before clean/aseptic procedure” (64.4%; 95%CI, 63.7–65.1%), “after touching a patient” (57.5%; 95%CI, 56.5–58.5%), “after touching patient surroundings” (52.2%; 95%CI, 50.3–54.1%), and “before patient contact” (47.4%; 95%CI, 46.4–48.4%). Trends towards improvement were significant for all hand hygiene indications (Fig. 2). Most hand hygiene opportunities were observed among midwifes (51.9%), followed by physicians (44.2%). Nursing assistants and students only contributed to a small proportion of hand hygiene opportunities (1.4 and 2.6%, respectively). Figure 3 shows the trends of hand hygiene compliance for the professional categories. Compliance was highest for physicians (60.8%; 95%CI, 60.1–61.5%), followed by midwifes (55.1%; 95%CI, 54.5–55.8%), and other professions (32.9%; 95%CI, 30.7–35.2%). Compliance improved significantly in all professional groups.

**Table 1:** Hand hygiene opportunities and compliance with either hand washing or alcohol-based hand rubbing – Hung Vuong Hospital, Ho Chi Minh City, Vietnam; 2010–2016

Year	All professions			Physicians			Midwifes			Other professions		
	N	HH	%	N	HH	%	N	HH	%	N	HH	%
2010	4079	877	21.5	1476	482	32.7	2449	388	15.8	154	7	4.5
2011	4482	1859	41.5	2505	1254	50.1	1891	601	31.8	86	4	4.7
2012	5477	2988	54.6	2555	1603	62.7	2252	1150	51.1	670	235	35.1
2013	4169	2227	53.4	1837	1161	63.2	1794	903	50.3	538	163	30.3
2014	4722	2548	54.0	2158	1132	52.5	2537	1402	55.3	27	14	51.9
2015	6179	3986	64.5	2623	1642	62.6	3436	2276	66.2	120	68	56.7
2016	5119	3325	65.0	1955	1311	67.1	3084	1964	63.7	80	50	62.5
2017	4333	3142	72.5	1929	1383	71.7	2375	1739	73.2	29	20	69.0
2018	5151	3865	75.0	2269	1776	78.3	2859	2081	72.8	23	8	34.8
Total	43,711	24,817	56.8	19,307	11,744	60.8	22,677	12,504	55.1	1727	569	32.9

*HH* hand hygiene action (through either alcohol-based hand rubbing, handwashing, or both)

**Fig. 4:**
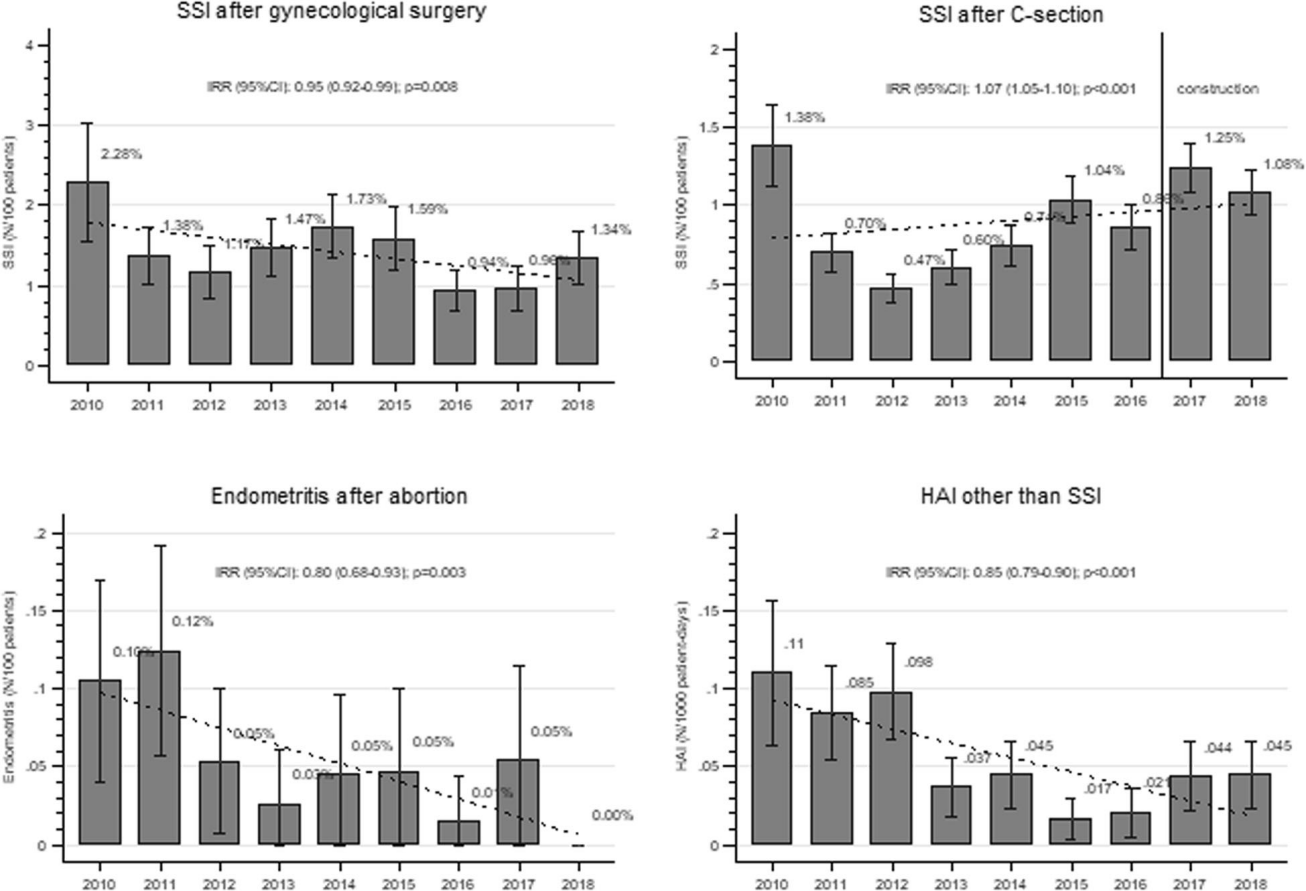
Trends of healthcare-associated infections – Hung Vuong Hospital, Ho Chi Minh City, Vietnam; 2010–2018. 95%CI: 95% confidence interval; HAI; healthcare-associated infection; IRR: incidence rate ratio; SSI: surgical site infection

Between 2010 and 2018, a total of 554,720 women were admitted for 353,919 deliveries (198,679 vaginal; 155,240 by C-section) and 257,127 surgical procedures, accumulating 3,150,793 patient-days. Figure 4 summarizes SSI and HAI trends. The overall incidence density of HAI decreased significantly from 1.10 episodes per 1000 patient-days in 2010 to 0.45 per 1000 patient-days in 2018 (IRR 0.85; 95%CI, 0.79–0.90). Significant decreasing trends of incidence proportions were observed also for SSI after gynaecological surgery (IRR 0.95; 95%CI, 0.92–0.99]) and endometritis after abortion (IRR 0.80; 95%CI, 0.68–0.93). After initial decrease, the incidence of SSI after C-section increased significantly over time (IRR 1.07; 95%CI, 1.05–1.10), particularly from 2017.

## Discussion

This observational, quasi-experimental intervention study shows the benefits of a prospective multimodal strategy enhancing hand hygiene compliance in gynaecology and obstetrics in a lower-middle-income country in Southeast Asia. Hand hygiene improved significantly from a low compliance in 2010 to reach 65% in 2015 and 75.1% in 2018. The high number of observed hand hygiene opportunities together with the prospective, continuous measurement of HAI over 9 years in a high-volume referral maternity hospital, makes this study unique and the largest in the field of hand hygiene in gynaecology and obstetrics to the best of our knowledge.

Only one study using a similar methodology was identified in the literature [18]. The publication addressed hand hygiene in a rural teaching hospital in Uganda, where only half of the patients were hospitalized in obstetrics. The size of the study was much smaller compared to our study, and the external setting of rural Uganda was different from ours of a large urban area. The findings of this study are representative for the situation of low-and-middle-income countries. The baseline incidence of SSI after gynaecological surgery in 2010 (2.3%; 95%CI,1.6–3.0) was similar to other low-and-middle-income countries [19], but higher compared to the USA [20]. Similarly, SSI after C-section in 2010 (1.4; 95%CI, 1.1–1.7]), and again in 2017 and 2018, was comparable to findings in Saudi Arabia and Brazil [21, 22], but higher compared to the findings of the European Centre for Disease Prevention and Control and the International Nosocomial Infection Control Consortium [19, 23]. Thus, although performed in a single centre, the results appear to be generalizable to the Asia Pacific region and other low-and-middle-income countries.

Both the average hand hygiene compliance before the multifaceted prevention programme and the observed improvement following successful hand hygiene promotion are consistent with earlier reports from low-and-middle-income [24] as well as from high-income countries [25–27]. Improvement was observed across all hand hygiene indications and the two major professional categories; and it was sustained. Only few studies demonstrated sustained hand hygiene improvement over more than 3 years [28–32]. In contrast to other findings, both in Vietnam and other countries, hand hygiene compliance among physicians was higher compared to midwifes [33]. However, hand hygiene compliance improved in both professional categories converging to similar levels from 2013 onwards. In another Vietnamese acute care hospital, the Hue central hospital, hand hygiene compliance among physicians was lower compared to nurses (34% [95%CI, 26–43%] and 57% [95%CI, 55–60%], respectively) [33]. The reason for the relatively high and sustained hand hygiene compliance among physicians in the current study cannot be fully established; importantly however, indicators showed a high-level institutional safety culture during the study period, including the support of the hospital management, established monitoring and performance feedback, and hygiene promotion through a variety of activities and events. The hand hygiene improvement programme not only had the oral and written support of the hospital director, but he was a role model for hand hygiene himself in daily practice. It is to note that this physician champion was the director of the IPC programme before becoming the hospital director.

In 2006, all HCWs at HVH received a 2-h training course on hand hygiene knowledge, which resulted in a short-lived increase of hand hygiene compliance to 32%. However, due to education targeting new employees only, compliance dropped to 8% 3 years later, similar to levels identified in other hospitals in Vietnam (e.g. 6.3% in the Bach Mai hospital in 2009) [34], and worldwide at that time [24, 25]. As recommended by WHO [13], and further proven through a meta-analysis [27], the current intervention was multimodal and sequential [2]. This strategy ensured ongoing exposure of the HCWs to a variety of original promotion activities. Similarly to previous reports [4, 24, 26, 35, 36], it is not possible to identify one particular element of the intervention to be responsible for the significant hand hygiene improvement. However, we consider that the following elements contributed to the observed positive outcome: 1) addressing all professional categories with direct patient contact; 2) use of all elements of the WHO multimodal promotion strategy and all implementation tools; 3) repeated interventions using different, and locally and timely adapted modes of education and training; 4) strong credible support by the hospital management; 5) provision of ABHR at every point of care; 6) and local production of ABHR in a country where acquisition of such products can be costly. As shown, hand hygiene improvement was largely due to hand rubbing, similar to other studies in Vietnam [37] and worldwide [24, 26, 38]. The shift from handwashing with soap and water to hand rubbing with ABHR most likely contributed to the success of the current intervention, as observed on other occasions before [39, 40]. Time constraint has been identified as one of the main risk factors for non-compliance with hand hygiene [41], and the preferred recourse to ABHR for action is one way to bypass such constraints and improve compliance [42]. In this regard, HVH was perfectly prepared to apply the WHO recommended multimodal strategy before the study. The strategy requires what is called a “system change” [38], which was implemented in the hospital before the implementation of the WHO strategy, as illustrated by the 1:1 ABHR dispenser-to-patient ratio.

Similar to other reports [3], the observed decrease of HAIs other than SSI over the years can be considered partially a result of the hand hygiene intervention. However, in addition to the ongoing hand hygiene promotion, a programme in 2012/2013 aiming at CAUTI prevention contributed to the observed drop of HAI in the following years, since CAUTI was the most frequent HAI other than SSI. The association between hand hygiene and HAI could not be tested in multivariable models, which limits such interpretation. Even if hand hygiene as a single intervention cannot explain the total of the outcome, multimodal prevention strategies together with surveillance and feedback can have effects on quality improvement by changing HCW behaviour on a more general level [43]. This is why the use of multimodal strategies is one of the key components of successful infection control [2, 44]. The decrease of SSI over time is due to specific prevention strategies aiming at improving surgical hand preparation and skin antisepsis. The increasing trend of C-section, reverse to all other surgical procedures, is most likely due to construction and relocation activities in this area, starting in July 2017.

The study has limitations: 1) patient data were collected for HAI cases only, which did not allow to analyse data in a multivariable model, controlling for intrinsic risk factors; the large sample size and studied population partly corrected for this issue though; 2) for the same reasons, the association between hand hygiene intervention and HAI reduction is weak; and although we can assume partial contribution to the positive outcomes, the trends of SSI after gynaecological surgery and abortion are more likely due to the combined effect of hand hygiene improvement and specific prevention strategies in surgery; 3) the multilevel and sequential character of the intervention does not allow to analyse the contribution of single elements of the intervention to the overall outcome. However, first, this is rather the rule than the exception in quality improvement studies aiming at behaviour change of HCWs [45], and, second, only multimodal promotion strategies revealed to improve hand hygiene behaviour, the more elements are included in the strategy, the larger is the impact [27].

In 2014, HVH has been one of the hospitals in the Asian-Pacific region receiving the “Hand Hygiene Excellence Award” (www.hhea.info), recognizing structure changes resulting from efforts to successfully promote and sustain good hand hygiene practice [46, 47]. Results presented here confirm the validity of the award selection process resulting in sustained levels of hand hygiene, and should stimulate healthcare institutions around the world to monitor their level of preparedness to hand hygiene promotion, improve it, and confront their level to the best examples.

## Conclusions

A multimodal strategy aiming at behaviour change significantly improved and sustained hand hygiene among physicians and midwifes in a large gynaecology and obstetrics hospital in a lower-middle-income country. Improved hand hygiene contributed to the reduction of healthcare-associated infections other than surgical-site.

## Data Availability

The datasets used and/or analysed during the current study are available from the corresponding author on reasonable request.

## Abbreviations

95%CI: 95% confidence interval
ABHR: Alcohol-based handrub
CAUTI: Catheter-associated urinary tract infection
CDC: US Centers for Disease Prevention and Control
HAI: Healthcare-associated infection
HH: Hand hygiene
HCW: Healthcare worker
HVH: Hung Vuong Hospital
IPC: Infection prevention and control
IQR: Interquartile range
IRR: Incidence rate ratio
STROBE: Strengthening the Reporting of Observational studies in Epidemiology
SSI: Surgical site infection
WHO: World Health Organization
